# Effects of Light Intensity on Growth and Quality of Lettuce and Spinach Cultivars in a Plant Factory

**DOI:** 10.3390/plants12183337

**Published:** 2023-09-21

**Authors:** Chen Miao, Shaojun Yang, Jing Xu, Hong Wang, Yongxue Zhang, Jiawei Cui, Hongmei Zhang, Haijun Jin, Panling Lu, Lizhong He, Jizhu Yu, Qiang Zhou, Xiaotao Ding

**Affiliations:** 1 Shanghai Key Laboratory of Protected Horticultural Technology, Horticulture Research Institute, Shanghai Academy of Agricultural Sciences, Jinqi Road No. 1000, Fengxian District, Shanghai 201403, China; miaochen@saas.sh.cn (C.M.); wanghong@saas.sh.cn (H.W.); xuezylemon@foxmail.com (Y.Z.); cuijiawei@saas.sh.cn (J.C.); zhanghongmei@saas.sh.cn (H.Z.); jinhaijun@saas.sh.cn (H.J.); lpl2245@163.com (P.L.); hlznd02@163.com (L.H.); yy2@saas.sh.cn (J.Y.); zhou.qiang@dushigreen.com (Q.Z.); 2 Shanghai Youyou Agricultural Technology Co., Ltd., Yuanqu South Road No. 1000, Chongming District, Shanghai 202150, China; yangshaojun@youyou.com.cn; 3 Department of Horticulture, Shanghai Institute of Technology, Haiquan Road No. 100, Fengxian District, Shanghai 201418, China; xujingenen@163.com

**Keywords:** indoor cultivation, LED light intensity, plant development, photosynthesis, nutrient content, tipburn

## Abstract

The decreased quality of leafy vegetables and tipburn caused by inappropriate light intensity are serious problems faced in plant factories, greatly reducing the economic benefits. The purpose of this study was to comprehensively understand the impact of light intensity on the growth and quality of different crops and to develop precise lighting schemes for specific cultivars. Two lettuce (*Lactuca sativa* L.) cultivars—Crunchy and Deangelia—and one spinach (*Spinacia oleracea* L.) cultivar—Shawen—were grown in a plant factory using a light-emitting diode (LED) under intensities of 300, 240, 180, and 120 μmol m^−2^ s^−1^, respectively. Cultivation in a solar greenhouse using only natural light (NL) served as the control. The plant height, number of leaves, and leaf width exhibited the highest values under a light intensity of 300 μmol m^−2^ s^−1^ for Crunchy. The plant width and leaf length of Deangelia exhibited the smallest values under a light intensity of 300 μmol m^−2^ s^−1^. The fresh weight of shoot and root, soluble sugar, soluble protein, and ascorbic acid contents in the three cultivars increased with the increasing light intensity. However, tipburn was observed in Crunchy under 300 μmol m^−2^ s^−1^ light intensity, and in Deangelia under both 300 and 240 μmol m^−2^ s^−1^ light intensities. Shawen spinach exhibited leaf curling under all four light intensities. The light intensities of 240 and 180 μmol m^−2^ s^−1^ were observed to be the most optimum for Crunchy and Deangelia (semi-heading lettuce variety), respectively, which would exhibit relative balance growth and morphogenesis. The lack of healthy leaves in Shawen spinach under all light intensities indicated the need to comprehensively optimize cultivation for Shawen in plant factories to achieve successful cultivation. The results indicated that light intensity is an important factor and should be optimized for specific crop species and cultivars to achieve healthy growth in plant factories.

## 1. Introduction

A plant factory is an environment-controlled plant production facility [1,2]. Based on the lighting systems, plant factories can be divided into three main types: (A) relying exclusively on artificial light (AL), (B) relying exclusively on natural light (NL), and (C) relying on a combination of NL and AL [3]. Type A involves a nearly fully enclosed building that uses various sensors and control software to precisely manage the growth environment of plants. This is commonly used for the commercial production of leafy greens and fruiting vegetables such as lettuce and tomatoes. Types B and C are advanced greenhouses that are not fully enclosed systems. The main difference between them is that in the case of a lack of sunlight, such as during the rainy season or winter, type C uses artificial lighting to prevent growth delays, yield reductions, and quality compromises [3]. Leafy vegetables are considered the most suitable crops for cultivation in plant factories due to their high yield and quality characteristics [4]. The AL source commonly used in plant factories is a light-emitting diode (LED), which has many advantages, including low energy consumption, long lifespan, and narrow spectral range [5].

Light plays a crucial role in the growth and development of plants, providing both energy and signal [6,7]. Light quality, intensity, and photoperiod are three important factors that affect plant growth [8]. Light intensity is an important factor that affects crop growth and quality [9]. It affects the morphology, photosynthetic characteristics, nutrient content, and antioxidant capacity of crops [10,11,12], which ultimately affect yield and quality. In addition, different species and cultivars respond differently to the same light intensity [13]. That is, for specific species or cultivars, precise lighting schemes are required to effectively improve plant yield and quality. Furthermore, such precise control of light intensity can reduce electricity consumption.

Light intensity influences the content of soluble sugar, soluble protein, and ascorbic acid. One crucial aspect of vegetable quality is taste, which is largely determined by the contents of soluble sugars and soluble protein [14,15,16]. These substances are responsible for providing characteristic flavor and texture to vegetables, and their accumulation within plants is highly dependent on light intensity [17]. A previous study demonstrated that light intensity plays a significant role in regulating the contents of glucose, fructose, ascorbic acid, and soluble sugar [18,19,20]. Moreover, exposure to appropriate light intensity during storage after harvesting is reported to be beneficial for the preservation of nutrients [21]. Given the importance of vegetable quality, suitable light intensity is a critical factor in promoting the accumulation of key nutrients and enhancing overall plant quality.

Inappropriate light intensity can create adverse conditions for plant growth. Photosynthesis is driven by the consumption of light energy. It is widely recognized that as the light intensity increases, the net photosynthetic rate (Pn) increases [22]. However, inappropriate light intensity may limit the carbon assimilation process and reduce Pn [23]. Therefore, plants do not use all the absorbed light for photosynthesis. When the absorbed light energy exceeds the photosynthetic capacity, plants release some of the energy in the form of heat. Moreover, excessive light intensity may damage plants [24,25], which is reflected by certain indicators such as tipburn. In addition, the photosynthetic capacity of plants is related to their variety-specific characteristics imparted by the differences in plant architecture, the leaf pigment pool, and stomata traits of different cultivars [25,26]. For example, the Pn of green lettuce is usually higher than that of red lettuce [27].

Different crops and cultivars have varying abilities to adapt to light environments. When fluorescent lamps were used as the light source, increasing the light intensity from 270 to 570 μmol m^−2^ s^−1^ significantly increased the fresh weight, dry weight, leaf thickness, and other indicators of Lollo Rosso lettuce. However, such effects were not observed when LED lights were used as the light source [28]. The adaptability of green and red lettuce to blue light differs among varieties. For example, the rosette fresh weight of red lettuce is 19–25% higher than that of green lettuce under blue light treatment [27].

Currently, one of the major challenges in the production of leafy vegetables in plant factories is setting up appropriate light intensities for different crops or varieties. This is because most studies focus on the effects of different light intensities on the same crop, and research on the response and performance of different crops and varieties to various light intensities is limited. Additionally, the response of different plant architectures to light environments needs to be studied. Therefore, we have set up four light-intensity gradients and selected one spinach (*Spinacia oleracea* L.) cultivar and two lettuce (*Lactuca sativa* L.) cultivars with different morphological characteristics to validate our hypothesis that higher light intensity promotes faster growth and a higher quality of lettuce and spinach, leading to increased yields. However, when light intensity exceeds a certain threshold, plants may exhibit stress responses. In this study, we comprehensively analyzed the impact of light intensity on the growth and development of these crops and cultivars, which will help in optimizing the delivery of high-quality products by designing an efficient lighting scheme.

## 2. Results

### 2.1. Plant Height, Plant Width, Number of Leaves, Leaf Length, Leaf Width, and Fresh Weight of Shoot and Root

**Figure 1:**
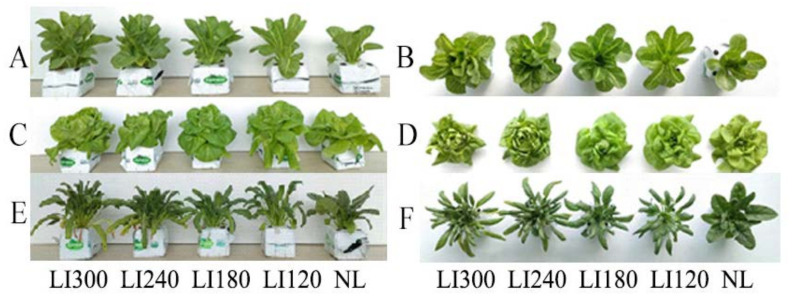
Lettuce cultivars Crunchy (**A**,**B**) and Deangelia (**C**,**D**) and spinach cultivar Shawen (**E**,**F**) grown in a solar greenhouse with natural light (NL) and a closed-type plant factory with light intensities of 300, 240, 180, and 120 μmol m^−2^ s^−1^ (indicated by LI300, LI240, LI180, and LI120, respectively).

**Figure 2:**
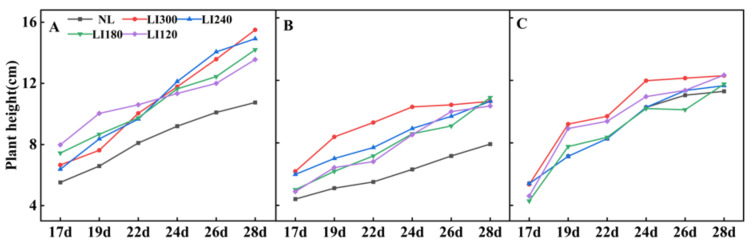
Plant height of two lettuce cultivars Crunchy (**A**) and Deangelia (**B**) and one spinach cultivar Shawen (**C**) grown in a solar greenhouse (NL) and a closed-type plant factory with light intensities of 300, 240, 180, and 120 μmol m^−2^ s^−1^ (indicated by LI300, LI240, LI180, and LI120, respectively).

Various growth parameters of the three cultivars were assessed under LED light intensities of 300, 240, 180, and 120 μmol m^−2^ s^−1^ (indicated by LI300, LI240, LI180, and LI120, respectively). Compared with NL (a solar greenhouse with natural light), cultivation under LED (a closed-type plant factory with LED light) clearly increased the plant height of Crunchy and Deangelia (Figure 1 and Figure 2). Under LED treatments, the plant height of the three cultivars exhibited different growth trends over time. For Crunchy, the maximum and minimum plant height was observed under LI120 and LI240 on the 17th day, respectively, and the plant height increased with increasing light intensity on the 28th day (Figure 1A and Figure 2A). For Deangelia, the plant height growth rate was higher under LI300 than under other LED light intensities from the 17th to 26th day. However, its plant height under the four LED light intensities was almost the same on the 28th day (Figure 1C and Figure 2B). For Shawen, on the 28th day, the plant height under LI300 and LI120 was similar and higher than those under LI240 and LI180, and the plant height was the shortest under NL (Figure 1E and Figure 2C). These results indicated that plant heights of different species and cultivars exhibited different responses to light intensity.

**Figure 3:**
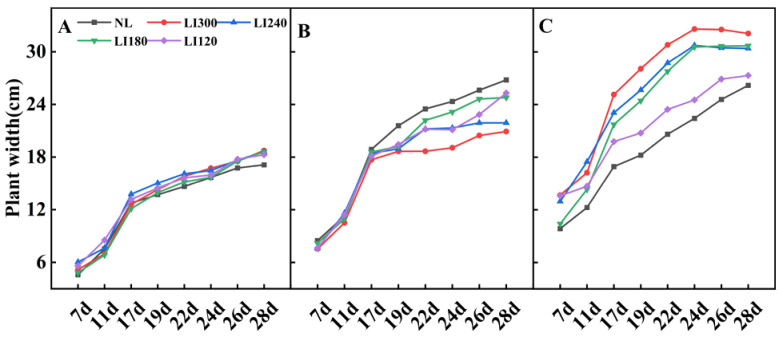
Plant width of two lettuce cultivars Crunchy (**A**) and Deangelia (**B**) and one spinach cultivar Shawen (**C**) grown in a solar greenhouse (NL) and a closed-type plant factory with light intensities of 300, 240, 180, and 120 μmol m^−2^ s^−1^ (indicated by LI300, LI240, LI180, and LI120, respectively).

Different LED light intensities exhibited different effects on the plant width of lettuce and spinach (Figure 1 and Figure 3). For Crunchy, the plant width rapidly increased under all LED treatments from the 7th to the 17th day, after which the growth rate slowed down. The plant width under LED was slightly larger than that under NL, and no considerable difference existed among different LED light intensities (Figure 1B and Figure 3A). For Deangelia, the plant width rapidly increased from the 7th to the 17th day under LED and NL, followed by a slower increase. On the 17th day, a larger plant width was exhibited under NL than under four LED light intensities. Among the LED light intensities, LI120 and LI300 exhibited the largest and smallest plant widths on the 28th day, respectively (Figure 1D and Figure 3B). For Shawen, the plant width rapidly increased from the 7th to the 24th day, after which the growth rate slowed down; the plant width under LED was clearly higher than that under NL (Figure 1F and Figure 3C). The rapid growth period of plant width was longer for Shawen than for both lettuce cultivars, which may be attributed to interspecific differences.

**Figure 4:**
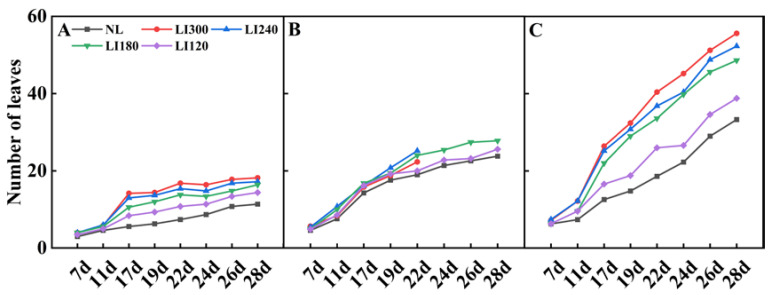
Number of leaves of two lettuce cultivars Crunchy (**A**) and Deangelia (**B**) and one spinach cultivar Shawen (**C**) grown in a solar greenhouse (NL) and a closed-type plant factory with light intensities of 300, 240, 180, and 120 μmol m^−2^ s^−1^ (indicated by LI300, LI240, LI180, and LI120, respectively).

**Figure 5:**
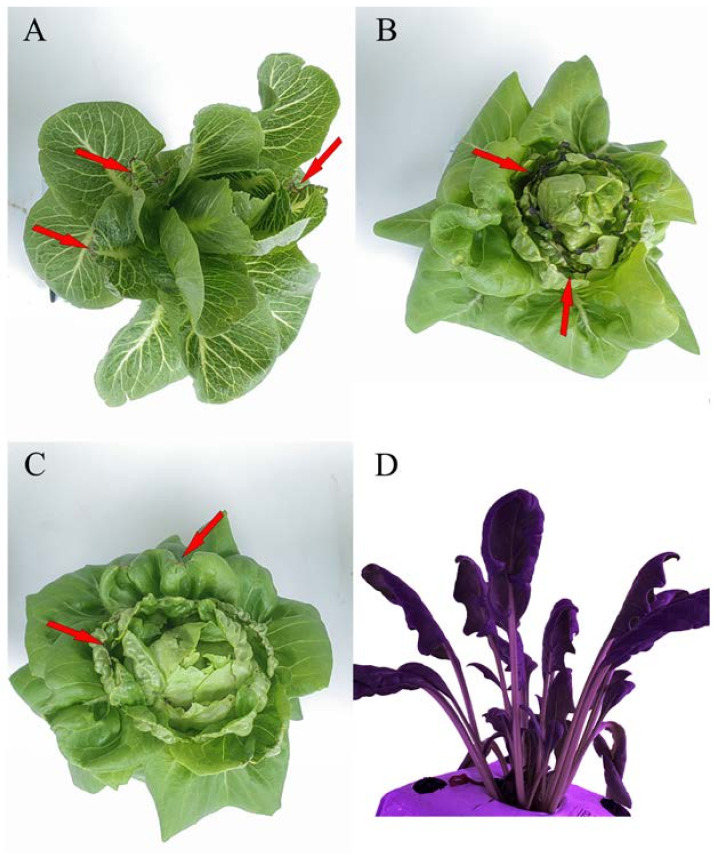
Tipburn in Crunchy under light intensity of 300 μmol m^−2^ s^−1^ (**A**) and in Deangelia under light intensities of 300 and (**B**) and 240 (**C**) μmol m^−2^ s^−1^. Red arrow points to the exact location of the tipburn. Leaf curling in Shawen (**D**).

The number of leaves under LED treatments was higher than that under NL (Figure 4). Interestingly, both Crunchy and Shawen demonstrated an increase in the number of leaves with increasing light intensity, indicating a positive correlation between light intensity and leaf development (Figure 4A,C). However, Deangelia exhibited some anomalies under high light intensity. Specifically, the inner leaves could not expand under LI300 and LI240 on the 22nd day, as it entered the heading stage (Figure 1D). Therefore, we stopped counting the number of leaves from the 22nd day. Nevertheless, under LI180 and LI120, Deangelia exhibited normal plant morphology. The number of leaves was higher under LI180 than under LI120 (Figure 4B). Importantly, the leaves of Crunchy exhibited tipburn under LI300 (Figure 5A), whereas Deangelia exhibited tipburn and leaf shrinkage under LI300 and LI240 (Figure 5B,C).

**Figure 6:**
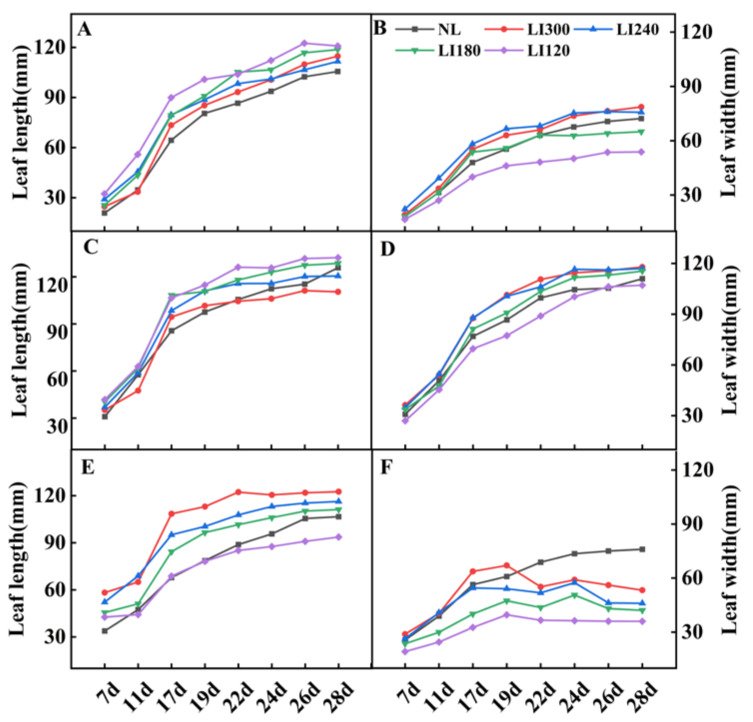
Leaf length and leaf width of two lettuce cultivars Crunchy (**A**,**B**) and Deangelia (**C**,**D**) and one spinach cultivar Shawen (**E**,**F**) grown in a solar greenhouse (NL) and a closed-type plant factory with light intensities of 300, 240, 180, and 120 μmol m^−2^ s^−1^ (indicated by LI300, LI240, LI180, and LI120, respectively).

For both lettuce cultivars, leaf length decreased (Figure 6A,C) but leaf width increased with increasing light intensity (Figure 6B,D). However, for Shawen, both leaf length and width were positively correlated with light intensity (Figure 6E,F). Additionally, under LED treatments, leaf curling occurred in Shawen on the 19th day (Figure 1E and Figure 5D); this led to a decrease in leaf width, which was smaller than that under NL (Figure 6F).

**Figure 7:**
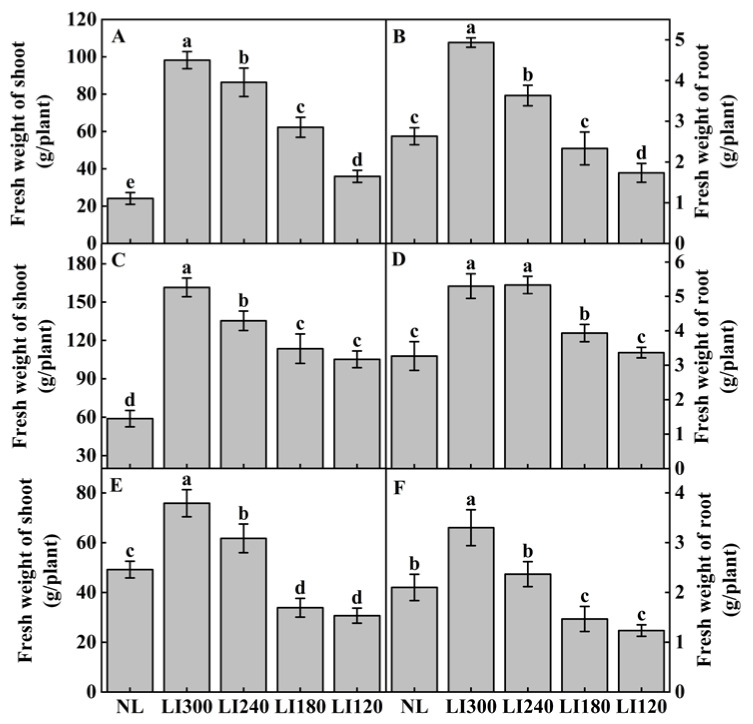
Fresh weight of shoot and root of two lettuce cultivars Crunchy (**A**,**B**) and Deangelia (**C**,**D**) and one spinach cultivar Shawen (**E**,**F**) grown in a solar greenhouse (NL) and a closed-type plant factory with light intensities of 300, 240, 180, and 120 μmol m^−2^ s^−1^ (indicated by LI300, LI240, LI180, and LI120, respectively). Bars with different letters within each panel indicate significant differences at *p* < 0.05 according to Duncan’s test.

The fresh weight of shoot and root increased with the increasing LED light intensity (Figure 7). The fresh weight of Crunchy was significantly different under four LED light intensities (Figure 7A,B). The maximum fresh weight of the shoot and root was observed at LI300, exhibiting a 306.9% and 77.8% increase compared with NL, respectively. However, no significant differences were observed between LI180 and LI120 in terms of the fresh weight of the shoots of Deangelia (Figure 7C) and Shawen (Figure 7E); the fresh weight of the shoots was significantly higher under LI300 in Deangelia and Shawen, exhibiting an increase of 174.3% (Figure 7C) and 54.2% (Figure 7E) compared with NL, respectively. For the two lettuce cultivars, the fresh weight of the shoots under LED was significantly higher than under NL (Figure 7A,C). The fresh weight of the shoot of Shawen was significantly higher than NL only under LI300 and LI240 (Figure 7E). The effect of different LED light intensities on the fresh weight of the root was comparable with the variation observed in the fresh weight of the shoot.

### 2.2. Leaf Photosynthetic Net Rate and Chlorophyll Fluorescence

For lettuce cultivars, leaf Pn increased with increasing light intensity (Table 1). For Deangelia, the Pn, intercellular CO_2_ concentration (Ci), stomatal conductivity (Gs), and transpiration (Tr) were significantly high under LED cultivation compared with NL. For Crunchy and Shawen, only when light intensity was higher than LI120, Pn was significantly higher than that under NL. The maximum Pn was observed at LI300 for lettuce cultivars. For Shawen, no significant difference was observed in Pn under LI300, LI240 and LI180. Notably, the water use efficiency (WUE) of all three cultivars was significantly higher under NL than under LED treatments.

**Figure 8:**
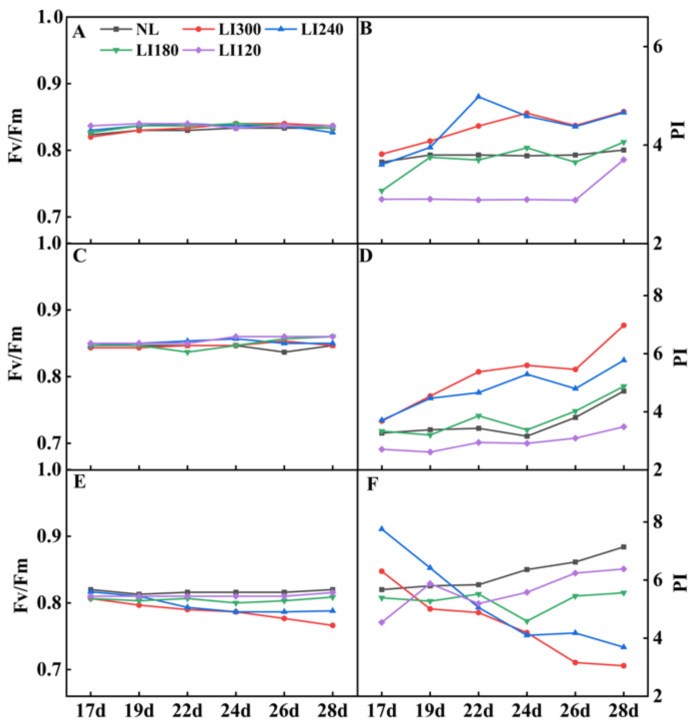
Fv/Fm and PI of two lettuce cultivars Crunchy (**A**,**B**) and Deangelia (**C**,**D**) and spinach cultivar Shawen (**E**,**F**) grown in a solar greenhouse (NL) and a closed-type plant factory with light intensities of 300, 240, 180, and 120 μmol m^−2^ s^−1^ (indicated by LI300, LI240, LI180, and LI120, respectively).

Fv/Fm values are widely used to study plant stress response, photosynthetic damage, and ecosystem productivity. The photosynthetic performance index (PI) is commonly used to study the response of plants to growth conditions in the environment, as it can indicate the health status of plant leaves and their photosynthetic efficiency. Under LED treatments, Crunchy and Deangelia exhibited no difference in Fv/Fm over time (Figure 8A,C). For both Crunchy and Deangelia, the PI value exhibited an upward trend over time, exhibiting the order of LI300 > LI240 > LI180 > LI120 on the 28th day (Figure 8B,D). In contrast, both the Fv/Fm and PI values of Shawen decreased over time under LI300 and LI240, whereas relatively stable trends or slow increases were displayed under LI180 and LI120 (Figure 8E,F).

### 2.3. Soluble Sugar, Soluble Protein, and Ascorbic Acid Contents

**Figure 9:**
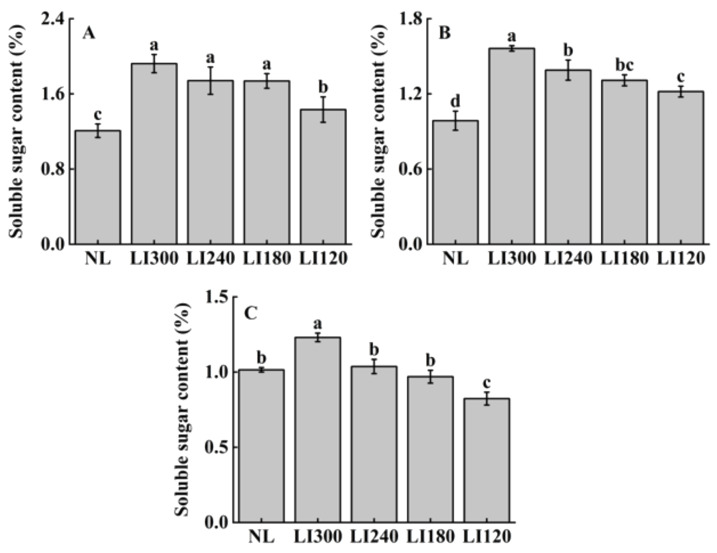
Soluble sugar content of two lettuce cultivars Crunchy (**A**) and Deangelia (**B**) and one spinach cultivar Shawen (**C**) grown in a solar greenhouse (NL) and a closed-type plant factory with light intensities of 300, 240, 180, and 120 μmol m^−2^ s^−1^ (indicated by LI300, LI240, LI180, and LI120, respectively). Bars with different letters within each panel indicate significant differences at *p* < 0.05 according to Duncan’s test.

The soluble sugar content positively correlated with light intensity for all three cultivars (Figure 9). Under LED treatments, all three cultivars exhibited the highest and lowest soluble sugar contents at LI300 and LI120, respectively, with significant differences between the two levels (Figure 9A–C). Compared with NL, both lettuce cultivars exhibited significantly higher soluble sugar content under all LED treatments (Figure 9A,B); however, Shawen exhibited higher soluble sugar content only under LI300, with a significantly lower content observed at LI120 (Figure 9C).

**Figure 10:**
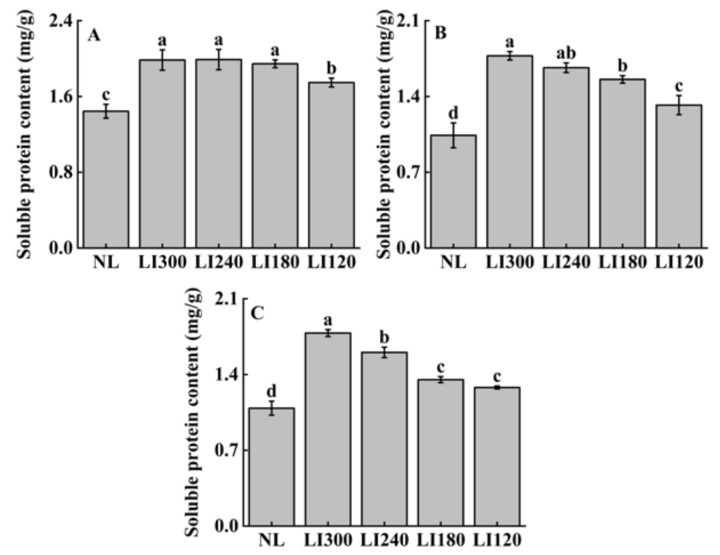
Soluble protein content of two lettuce cultivars Crunchy (**A**) and Deangelia (**B**) and one spinach cultivar Shawen (**C**) grown in a solar greenhouse (NL) and a closed-type plant factory with light intensities of 300, 240, 180, and 120 μmol m^−2^ s^−1^ (indicated by LI300, LI240, LI180, and LI120, respectively). Bars with different letters within each panel indicate significant differences at *p* < 0.05 according to Duncan’s test.

Figure 10 indicates the varietal specificity in terms of soluble protein content at different light intensities. Among all LED treatments, the soluble protein content was similar under LI300, LI240, and LI180 for Crunchy, and it was significantly higher than that under LI120 (Figure 10A). For Deangelia and Shawen, soluble protein content increased with increasing light intensity, with significantly higher content observed at LI300 than at LI180 and LI120 (Figure 10B,C). Compared with NL, all three cultivars exhibited significantly higher soluble protein content under LED treatments (Figure 10A–C).

**Figure 11:**
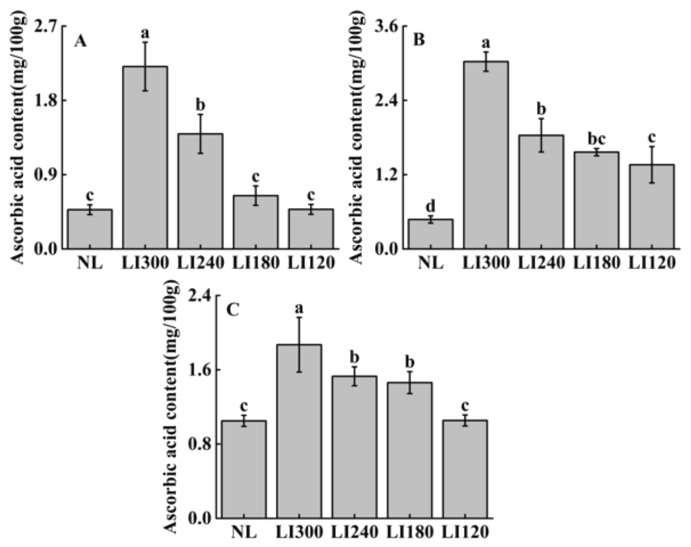
Ascorbic acid content of two lettuce cultivars Crunchy (**A**) and Deangelia (**B**) and one spinach cultivar Shawen (**C**) grown in a solar greenhouse (NL) and a closed-type plant factory with light intensities of 300, 240, 180, and 120 μmol m^−2^ s^−1^ (indicated by LI300, LI240, LI180, and LI120, respectively). Bars with different letters within each panel indicate significant differences at *p* < 0.05 according to Duncan’s test.

Ascorbic acid contents varied at different LED light intensities in the three cultivars (Figure 11). Crunchy exhibited significantly higher ascorbic acid content at LI300 than at LI240, LI180, and LI120, with increases of 157.9%, 191.8%, and 197.9%, respectively (Figure 11A). Deangelia exhibited significantly higher ascorbic acid content at LI300 among all LED treatments, whereas no significant difference was observed between LI240 and LI180 in terms of ascorbic acid content (Figure 11B). The pattern of variation in ascorbic acid content was similar in Shawen and Deangelia, with significantly higher content under LI240 and LI180 than under LI120 (Figure 11C). Compared with NL, both lettuce cultivars exhibited significantly higher ascorbic acid content under all LED treatments (Figure 11A,B); however, Shawen exhibited significantly higher ascorbic acid content under light intensities higher than LI180 (Figure 11C).

## 3. Discussion

The parameters of light have complex effects on photosynthesis and the quality and yield of plants [25,29]. This emphasizes the importance of optimizing lighting in plant cultivation. This is particularly important for plants grown under AL, such as lettuce, where the regulation of light parameters is crucial. In this study, increasing the light intensity positively affected the plant height and leaf numbers in two lettuce cultivars and one spinach cultivar (Figure 2 and Figure 4), which is consistent with previous studies [30,31]. Interestingly, the plant width of Crunchy was not affected by varying light intensities, whereas that of Deangelia decreased as light intensity increased (Figure 3A). However, the plant width of Shawen positively correlated with light intensity (Figure 3C). These results highlighted the influence of species-specific traits, such as morphological and physiological characteristics (e.g., plant structure and stomatal features) [27,32], making certain cultivars more suitable or less suitable for specific environments. Deangelia is a semi-heading lettuce variety, and the leaves of this cultivar exhibited leaf shrinkage under LI300 and LI240 (Figure 5), which resulted in a decrease in plant width (Figure 3B). This symptom may be due to excessive light intensity, as well as species-specific traits.

In addition, tipburn was observed in Crunchy and Deangelia (Figure 5). Tipburn not only reduces the quality and yield of the products but also decreases the marketable rate. Tipburn is a serious problem in the indoor cultivation of leafy vegetables [33]. Previous studies reported that the main reason for tipburn is calcium deficiency at the leaf tips, caused by rapid growth under high light intensity [34,35,36]. Plants exhibit higher photosynthetic rates under higher light intensities (Table 1), resulting in faster growth. However, the rapidly growing inner leaves were unable to absorb sufficient calcium and translocate it to the leaf tips, which led to tipburn [35]. Furthermore, during the rapid growth period of plants, more calcium is transferred to the outer leaves rather than the inner enclosed leaves [30]. Therefore, tipburn often occurs in the inner leaves. It is possible to alleviate tipburn by spraying calcium salt solution on the inner leaves [30]. Further comparing Crunchy and Deangelia, Crunchy exhibited a non-heading trait and Deangelia is a semi-heading lettuce. Both have completely different plant structures. Therefore, when Deangelia enters the heading stage, its inner leaves cannot expand fully. This may be the reason why the incidence of tipburn was higher in Deangelia than in Crunchy.

To address the issue of tipburn, we note that breeding is more important and efficient than modifying cultivation methods. Ryder and Waycott [37] and SW Jang [38] developed the tipburn-resistant lettuce varieties Tiber and Sambokhacheong, respectively. However, breeding tipburn-resistant varieties specifically for plant factories is extremely difficult. As mentioned earlier, tipburn in lettuce is caused by rapid growth, and plant factories and farmers want to cultivate lettuce that grows quickly to achieve better economic benefits [39]. This contradiction poses a great challenge for breeding. Plant factories can create a stable environment throughout the year, which speeds up the breeding process. Moreover, with in-depth research into the relationship between tipburn and genome [40], Japanese breeders have successfully bred tipburn-resistant lettuce varieties for plant factories. Increasing the number of tipburn-resistant lettuce varieties can promote large-scale lettuce production in plant factories. Our results (Figure 4 and Figure 6) confirmed that lower light intensities led to an elongated leaf blade and a reduced leaf number in the lettuce [41,42]. In contrast, Shawen displayed maximum and minimum leaf length and width at LI300 and LI120, respectively (Figure 6). This is because of the species-specific traits of the response to light intensity. Leaf structure reflects how environmental factors influence plants or how plants adapt to the changing environment [43]. In this study, after the 19th day of cultivation under LED treatments, significant leaf curling was observed in Shawen (Figure 1E,F). This was considerably different from its morphology under NL. This indicated that the environment in the plant factory in this experiment was not suitable for the growth of Shawen. In previous studies, the relative humidity (RH) in indoor cultivation experiments on spinach fluctuated between 45% and 80% [19,44]. However, in this study, the RH in the plant factory was 60–90%. Therefore, RH may be an important environmental factor affecting the leaf curling in Shawen. Therefore, appropriately reducing the RH in the plant factory may be an effective method to alleviate or eliminate leaf curling in Shawen. However, this should be further validated in future studies.

To achieve optimal yields and quality of different crop cultivars, it is essential to develop appropriate lighting schemes that cater to the unique demands of individual plants, thereby enhancing their productivity while reducing energy consumption. Our results (Figure 7) confirmed that low light intensity reduced the fresh weight of the shoot and root [42,45,46]. Under low light conditions, leaf chlorophyll content, Gs, and the activity of photosynthesis-related enzymes decrease [47,48]. Because of this, plants cannot perform adequate photosynthesis [49,50], which prevents organic matter accumulation and ultimately reduces both yield and quality. Importantly, in this study, the two lettuce cultivars displayed a higher fresh weight of shoot and root under LED treatments than under NL (Figure 7). This could be attributed to the higher emphasis on providing an optimal light scheme (light intensity and photoperiod) and temperature conditions in plant factories than in greenhouses during the winter season (Table 2), resulting in better plant yield and growth.

The magnitude of Pn can be used to assess the suitability of different light intensities for plants. It is generally acknowledged that low light conditions can inhibit photosynthesis to a certain extent [51]. Previous studies have reported that light intensity can affect Pn by altering the levels of enzymes involved in the carbon reduction cycle, electron transport components, and light-harvesting pigments [52,53,54]. In this study, high light intensity significantly enhanced the Pn, compared with low light intensity in lettuce cultivars (Table 1). This is consistent with a previous study by Fan [45]. Leaves are regarded as the primary organ for photosynthesis and transpiration. Under low light intensity, lettuce exhibited decreased leaf width (Figure 6). This, along with thinner leaf blades [55,56], may ultimately lead to a reduction in chloroplast number [57] and a subsequent decline in enzyme activity during the carbon cycle [52]. This may explain the decrease in Pn under low light intensity observed in this study (Table 1). Furthermore, the Pn of the three cultivars grown in the plant factory was higher or significantly higher than that of the plants grown in the greenhouse (Table 1). This may be due to a lower temperature [58,59], a shorter photoperiod [60], and a lower light intensity [22,31] in greenhouses than in plant factories in winter (January and February). These results further highlighted the superiority and necessity of the production of plants in plant factories during the winter season.

Agricultural products are dynamic composites of their physicochemical properties and evolving consumer perception, and they encompass organoleptic, nutritional, and bioactive components [61]. In all three cultivars, soluble sugar, soluble protein, and ascorbic acid contents were higher (significantly higher in Deangelia and Shawen, Shawen, and all three cultivars, respectively) under LI300 than under other LED light intensities (Figure 9, Figure 10 and Figure 11). These results confirmed that plants exhibit higher Pn under higher light intensity [62], which is beneficial for the accumulation of nutrients. This is consistent with previous studies [63,64]. In addition, soluble sugar, soluble protein, and ascorbic acid contents of the three cultivars were higher or significantly higher under LED than under NL. This is because the light, temperature, and RH can be reasonably regulated under LED treatments [65], which is more conducive to photosynthesis and more suitable for the accumulation of nutrients in plants. Importantly, all these results indicated the advantages of using plant factories over solar greenhouses in terms of plant production in winter.

## 4. Materials and Methods

### 4.1. Materials and Treatment Conditions

The experiment was conducted in a fully enclosed plant factory and an advanced solar greenhouse at Youyou Agricultural Technology Co., Ltd., Chongming District, Shanghai, China. The experimental materials were two lettuce cultivars (Crunchy and Deangelia) with different plant architectures and leaf shapes and one spinach cultivar (Shawen). On 23 December 2022, the seeds of the three cultivars were sown in coconut fiber blocks (10 cm length × 10 cm width × 7 cm height; Van der Knaap, Kwintsheul, the Netherlands) and germinated in a solar greenhouse until the two-leaf stage. On 3 January 2023, some of the seedlings were transferred to the fully enclosed plant factory for treatment with different LED light intensities. The remaining plants continued growing in the solar greenhouse with ambient average temperature and light intensity (Table 2). The temperature in the plant factory was maintained at 22 ± 2 °C, RH was 60–90%, and CO_2_ concentration was approximately 400 μmol mol^−1^. During the experiment, all plants were irrigated with nutrient solution (pH 5.8–6.5 and EC 1.7 mS cm^−1^) every 2–3 days.

**Figure 12:**
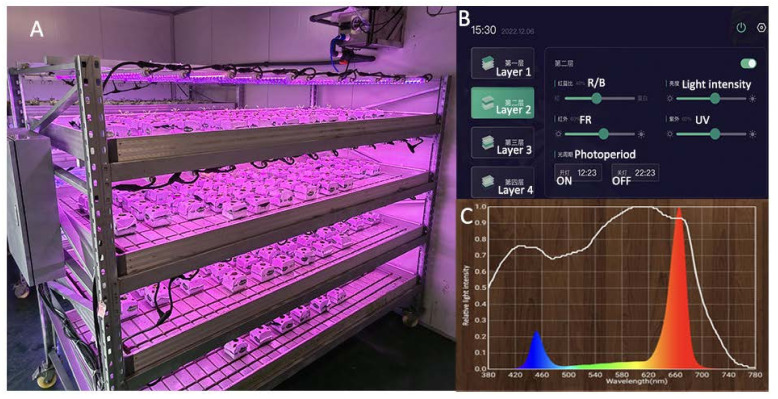
(**A**) The supplemental LED lighting system for plants. (**B**) Computer-based control system (**C**) The illumination spectrum used for LED treatments.

A specially designed plant supplement lighting system was used as a light source in the plant factory (Figure 12A). The system consisted of 4 layers, with 12 LEDs (Shanghai Sansi Electronic Engineering Co., Ltd., Shanghai, China) installed in each layer. The LED light tubes had a length of 1214 mm and a diameter of 30 mm. The peak wavelength for red and blue light was 666 nm and 452 nm, respectively, and the ratio of red (R) to blue (B) was 4 to 1. By integrating an advanced computer-based control system (Figure 12B), light intensity, light quality, and photoperiod could be precisely regulated. This enabled the control and manipulation of these parameters, ensuring optimal conditions for experimental purposes.

The experiment consisted of five treatments, as follows. Treatment 1 involved cultivation in a solar greenhouse using only NL with an average day/night temperature of 19.8/15.7 °C, average light intensity of 97 μmol m^−2^ s^−1^, and average photoperiod (day/night) of approximately 10 h/14 h (Table 2). Treatments 2 to 5, respectively, involved exposure to LED light intensities of 300, 240, 180, and 120 μmol m^−2^ s^−1^ (indicated by LI300, LI240, LI180, and LI120, respectively). The light intensity at the plant canopy was measured using the Lighting Passport instrument (Model No. ALP-01, Shanghai Hesheng Instrument Technology Co., Ltd., Shanghai, China) in conjunction with the Spectrum Genius APP (Asensetek Canada Inc., Quebec, Canada). Without intervention, the light intensity received by the plant changes as the plant grows taller; therefore, the computer-based LED plant supplementary lighting control system was adjusted to match the desired experimental light intensity after the measurement of growth parameters. The photoperiod was set to 16 h. A detailed illustration of the spectra for each LED treatment is presented in Figure 12C. Each light treatment contained 24 plant replicates in 3 plots, with 8 plants in each plot.

**Table 2:** Plant growth conditions under LED treatments.

Treatment	Temperature (°C) (Day/Night)	Light Intensity (μmol m^−2^ s^−1^)	Photoperiod (Day/Night)
NL	19.8/15.7	97	10 h/14 h
LI300	22 ± 2	300	16 h/8 h
LI240		240	16 h/8 h
LI180		180	16 h/8 h
LI120		120	16 h/8 h

### 4.2. Sample Collection

All plants were harvested on 3 February 2023. The fresh weight of each plant was measured immediately after harvest. During sample collection, five individual plants of each species were randomly selected as replicates for each treatment. Further, five plants were immediately homogenized using a blender, transferred to test tubes, frozen in liquid nitrogen, and stored at −40 °C for further analysis.

### 4.3. Assessment of Various Growth Parameters

#### 4.3.1. Plant Growth

Stem diameter, leaf length, and leaf width were measured using a vernier caliper. Plant width was measured using a ruler on the 7th, 11th, 17th, 19th, 22nd, 24th, 26th, and 28th days after the start of treatment. Leaf number was measured at the same time. Leaf length and width were measured using the largest fully expanded leaf, whereas plant width was measured at the widest point of the plant. The measurements of plant height were initiated from the 17th day. Plant height was determined from the base of the stem to the highest point on the plant using a ruler. Five biological replicates were measured for each treatment.

#### 4.3.2. Leaf Photosynthetic Rate and Chlorophyll Fluorescence

Photosynthetic parameters were determined using a CIRAS-3 portable photosynthesis system (PP Systems, Amesbury, MA, USA) on the 28th day. Prior to the measurements, samples were allowed to acclimatize to the measurement conditions for at least 30 min. Leaves were placed in a 4.5 cm^2^ conditioning chamber, where a light source provided a constant photon flux density of 1000 μmol m^−2^ s^−1^, to determine the Pn, Ci, Gs, Tr, and WUE. The values were recorded once the numerical fluctuations of the parameters were attenuated or reached a steady state. The photosynthetic parameters were calculated and presented as mean ± standard deviation (SD). Five biological replicates were measured for each treatment. The Fv/Fm and PI of leaves were determined using a Pocket Plant efficiency analyzer (PEA) (Hansatech Instruments Ltd., King’s Lynn, UK) from the 17th to 28th days. Prior to the measurements, leaves were adapted to the dark with a leaf clip holder for 30 min to achieve stable fluorescence emission. Five biological replicates were measured for each treatment.

#### 4.3.3. Soluble Sugar Content

Soluble sugar content was measured according to the method described previously [66]. A homogenized fresh leaf sample (0.5 g) was placed in a glass tube and supplemented with an appropriate amount of distilled water. The mixture was subsequently boiled for 30 min in a boiling water bath. The supernatant was filtered, and its volume was made up to 50 mL. The filtrate (0.5 mL) was mixed with 1.5 mL anthrone–ethyl acetate (analytical reagent (AR); Sinopharm Chemical Reagent Co., Ltd., Shanghai, China) solution and 1.5 mL distilled water. The mixture was treated with 5 mL sulfuric acid (AR, Sinopharm Chemical Reagent Co., Ltd., Shanghai, China) and heated for precisely 1 min in a boiling water bath, which resulted in the formation of a green solution. Once the solution cooled down to room temperature, the absorbance of the solution was measured at 630 nm using a UV-Vis spectrophotometer (Shimadzu Corporation, Kyoto, Japan). Three biological replicates were measured for each treatment.

#### 4.3.4. Soluble Protein Content

Soluble protein content was determined using the Coomassie brilliant blue G-250 method [66]. A homogenized fresh leaf sample (0.5 g) was mixed with 1.25 mL distilled water and centrifuged at 4 °C and 12,000× *g* for 20 min. The supernatant with the extracted proteins was collected. The supernatant (0.5 mL) was mixed with 5 mL Coomassie brilliant blue G-250 (BioReagent; SIGMA-ALDRICH Co., Saint Louis, MO, USA) solution and incubated for 2 min. Further, its absorbance was measured at 595 nm using a UV-Vis spectrophotometer (Shimadzu Corporation, Kyoto, Japan). Three biological replicates were measured for each treatment.

#### 4.3.5. Ascorbic Acid Content

Ascorbic acid content was determined using the acid–base titration method [66]. A homogenized fresh sample (5 g) was mixed with 20 g/L oxalic acid (guaranteed reagent (GR); Sinopharm Chemical Reagent Co., Ltd., Shanghai, China) solution until a volume of 50 mL was obtained. An appropriate amount of activated carbon was added to the mixture, and the mixture was filtered. The filtrate (10 mL) was transferred to a flask and titrated using a standardized 2,6-dichloroindophenol sodium salt (BioReagent, SIGMA-ALDRICH Co., Saint Louis, MO, USA) solution. The endpoint was reached when the solution turned light red and the color persisted for a duration of at least 15 s. Three biological replicates were measured for each treatment.

### 4.4. Data Analysis

All treatments were measured at least three times to ensure the accuracy of the data. Mean values and standard errors were calculated using Microsoft Excel version Mondo 2016 (Microsoft, Redmond, WA, USA). Duncan’s test was performed to determine the significance of mean differences (*p* < 0.05) using SPSS software version 22.0 (IBM Corporation, New York, NY, USA). The figures were drawn using Origin version 2022 (OriginLab Corporation, Northampton, MA, USA).

## 5. Conclusions

The photosynthetic rate, leaf width, fresh weight, soluble sugar, soluble protein, and ascorbic acid contents of both lettuce cultivars increased with the increasing LED light intensity. However, Crunchy (exhibiting the non-heading trait) developed tipburn when exposed to the light intensity of 300 μmol m^−2^ s^−1^, whereas tipburn and leaf shrinkage were observed in Deangelia (semi-heading lettuce cultivar) under both 240 μmol m^−2^ s^−1^ and 300 μmol m^−2^ s^−1^. The spinach cultivar, Shawen, exhibited leaf curling under all LED light intensities, impeding normal growth. These results indicated that although higher light intensity is beneficial for increasing yield and quality, the light intensity should be regulated as per the specific variety. Therefore, the optimum light intensities for Crunchy and Deangelia were 240 and 180 μmol m^−2^ s^−1^, respectively. However, under the experimental conditions in this study, Shawen spinach was observed to be not suitable for cultivation in a plant factory, Further studies are required to optimize the light conditions for the successful cultivation of this variety. Crunchy and Deangelia are two types of lettuce with different leaf shapes and plant architectures, and non-heading lettuce has higher adaptability to light intensity than semi-heading lettuce in plant factories. Crunchy is more suitable for cultivation and production in plant factories. We believe that this study provides additional insights into the selection of leafy vegetable varieties and lighting strategies in plant factories for their optimal production.

## Figures and Tables

**Table 1:** Photosynthetic parameters of two lettuce cultivars Crunchy and Deangelia and one spinach cultivar Shawen grown in a solar greenhouse (NL) and a closed-type plant factory with light intensities of 300, 240, 180, and 120 μmol m^−2^ s^−1^ (indicated by LI300, LI240, LI180, and LI120, respectively).

	Treatments	Pn (µmol CO_2_·m^−2^·s^−1^)	Ci (µmol CO_2_·mol^−1^)	Gs (mmol H_2_O·m^−2^·s^−1^)	Tr (mmol H_2_O·m^−2^·s^−1^)	WUE (mmol CO_2_·mol^−1^·H_2_O)
Crunchy	NL	7.8 ± 0.9 d	144.7 ± 6.7 b	47.0 ± 4.0 e	0.9 ± 0.1 c	8.7 ± 0.2 a
	LI300	21.3 ± 0.4 a	356.7 ± 2.5 a	436.3 ± 20.1 a	3.6 ± 0.1 a	5.9 ± 0 b
	LI240	18.4 ± 0.4 b	363.3 ± 4.2 a	353.3 ± 24.5 b	3.3 ± 0.1 a	5.6 ± 0.1 b
	LI180	11.5 ± 0.1 c	340 ± 14.1 a	144.3 ± 18.2 c	2.0 ± 0.2 b	5.9 ± 0.5 b
	LI120	7.8 ± 1.0 d	345 ± 33.8 a	99.0 ± 37.3 d	1.5 ± 0.6 b	5.4 ± 1.3 b
Deangelia	NL	7.3 ± 1.5 d	167.0 ± 3.0 c	47.0 ± 8.9 d	0.8 ± 0.1 c	9.1 ± 0.2 a
	LI300	23.0 ± 2.6 a	342.7 ± 19.9 b	395.3 ± 18.6 a	3.8 ± 0.6 b	6.1 ± 0.6 b
	LI240	20.5 ± 0.6 ab	338.3 ± 47.4 b	375.0 ± 9.8 ab	4.0 ± 1.1 b	5.3 ± 1.4 bc
	LI180	18.6 ± 0.6 b	407.0 ± 1.0 a	350.0 ± 29.8 b	5.3 ± 0.2 a	3.5 ± 0 d
	LI120	12.9 ± 1.2 c	376.0 ± 2.6 ab	301.3 ± 29.8 c	3.0 ± 0.1 b	4.3 ± 0.2 cd
Shawen	NL	10.0 ± 1.0 b	124.7 ± 4.0 c	50.0 ± 11.5 d	0.79 ± 0.2 c	11.5 ± 0.2 a
	LI300	13.6 ± 1.1 a	420.3 ± 9.5 ab	387.3 ± 21.5 a	5.3 ± 0.1 a	2.6 ± 0.2 c
	LI240	14.7 ± 1.9 a	416.3 ± 7.0 ab	345.3 ± 25.5 ab	5.4 ± 0.1 a	2.7 ± 0.3 c
	LI180	16.0 ± 0.7 a	441.7 ± 7.1 a	331.7 ± 19.8 b	5.5 ± 0.2 a	2.9 ± 0.1 bc
	LI120	10.1 ± 1.3 b	394.3 ± 34.1 b	243.3 ± 34.6 c	2.8 ± 0.7 b	3.8 ± 1.2 b

Different letters indicate significant differences (Duncan’s test at *p* < 0.05).

## Data Availability

The data presented in this study are available upon request from the corresponding author.
